# Impact of Anticholinergic Medication Burden on Mobility and Falls in the Lifestyle Interventions for Elders (LIFE) Study

**DOI:** 10.3390/jcm9092989

**Published:** 2020-09-16

**Authors:** Patrick Squires, Marco Pahor, Todd M. Manini, Scott Vouri, Joshua D. Brown

**Affiliations:** 1 Department of Pharmaceutical Outcomes & Policy, University of Florida College of Pharmacy, Gainesville, FL 32611, USA; patrick.squires@ufl.edu (P.S.); svouri@cop.ufl.edu (S.V.); 2 Center for Drug Evaluation & Safety, University of Florida, Gainesville, FL 32610, USA; 3 Institute on Aging, Department of Aging and Geriatric Research, University of Florida College of Medicine, Gainesville, FL 32610, USA; mpahor@ufl.edu (M.P.); tmanini@ufl.edu (T.M.M.)

**Keywords:** anticholinergic burden, mobility, falls, physical activity, successful aging

## Abstract

Anticholinergic cognitive burden (ACB) may be associated with detrimental effects on mobility and physical independence in older adults. We evaluated the incidence of major mobility disability (MMD), persistent major mobility disability (PMMD), and injurious falls among participants within the Lifestyle Interventions for Elders (LIFE) trial according to varied anticholinergic burden levels. Participants aged 70–89 years were randomized to a physical activity (PA) or successful aging (SA) intervention and evaluated by ACB medication use as a summed score of a previously developed ACB scale. Confounders included demographic characteristics, physical function, cognitive function, and fall history. Average participant follow-up was 2.6 years and included outcome assessment for MMD, PMMD, and injurious falls every six months. Adjusted proportional hazards models evaluated the independent effects of ACB scores as well as interaction effects with the intervention. Of the 1635 participants, 986 (60%) used ≥1 anticholinergic medication. Compared to those with no burden, participants with an ACB score of 1 demonstrated increased MMD (HR = 1.42 [1.13–1.78]), PMMD (HR = 1.53 [1.12–2.09]), and injurious falls (HR = 1.60 [1.10–2.32]). Results similar in magnitude were observed for all other ACB levels versus the no burden group. Stepwise dose–response comparisons between ACB groupings did not demonstrate significant differences in outcomes. Stratification by PA or SA interventions demonstrated few differences from the combined overall trial results. Compared to those not taking anticholinergic medications, participants taking anticholinergic medications generally demonstrated increased risk of MMD, PMMD, and injurious falls. Total anticholinergic burden was not associated with a stepwise dose–response relationship in mobility disability and may lack sensitivity to capture varied responses.

## 1. Introduction

The prescribing of anticholinergic medications in the older adult population has been deemed inappropriate due to the associated spectrum of central nervous system adverse effects including dizziness, sedation, confusion, and delirium, all of which contribute to a decline in both cognitive and physical function [1]. Multiple studies have examined the effect of anticholinergic burden on both cognitive and physical functioning domains with most studies in agreement of detrimental effects [2,3,4,5]. The Anticholinergic Cognitive Burden (ACB) scale is a medication scoring system used to predict the risk of anticholinergic adverse effects in older patients [6].

Little is known regarding the comparative effects of differing anticholinergic burdens paired with a physical activity intervention to help prevent mobility disability. A physical activity intervention may cancel out negative effects of increased anticholinergic burden by increasing participant strength and overall mobility outcomes or, conversely, anticholinergic medications may mediate response to physical activity. The objective of the present study was to evaluate the relationship between ACB and incidence of major mobility disability (MMD), persistent major mobility disability (pMMD), and injurious falls among participants in the Lifestyle Interventions for Elders (LIFE) study. Due to known detrimental anticholinergic effects on the older adult population, we hypothesized that total ACB would have a deleterious effect on MMD, pMMD, and injurious falls outcomes and in addition that the physical activity intervention may negate negative effects of those with high ACBs.

## 2. Methods

### 2.1. LIFE Study Overview

The LIFE Study was a multicenter, single-blind, parallel-assignment, randomized study conducted across eight centers in the United States between February 2010 and December 2013. The original LIFE study protocol was approved by respective institutional review boards at each participating institution. Written informed consent was obtained from all study participants. The trial was monitored by a data and safety monitoring board appointed by the National Institute on Aging. The LIFE study was registered with www.clinicaltrials.gov before participant enrollment in the trial (NCT01072500). The original LIFE study design, justification, and characteristics of the original study population have been previously described in detail [7,8]. Briefly, the inclusion criteria of the trial required that participants be between 70 and 89 years of age, score <10 on the Short Physical Performance Battery (SPPB), be sedentary with ≤125 min of activity per week, and be able to complete the 400 m walk test within 15 min without sitting, leaning, or assistance.

### 2.2. Interventions

The specific details of the study interventions of LIFE participants were published previously [7]. Enrolled subjects were asked to continuously participate in the study interventions and were ultimately followed for an average of 2.6 years. The physical activity (PA) intervention involved walking, with a goal of 150 min per week, in addition to strength, flexibility, and balance training. The PA intervention included attendance at two institutional visits per week and home-based activity three to four times per week for the duration of the study. The PA sessions were individualized and progressed toward a goal of 30 min of walking daily at moderate intensity, 10 min of primarily lower-extremity strength training by means of ankle weights (2 sets of 10 repetitions), 10 min of balance training, and large muscle group flexibility exercises.

The successful aging (SA) intervention comprised weekly educational workshops during the first 26 weeks with monthly sessions thereafter. Workshops included topics relevant to older adults, such as how to effectively negotiate the health care system, how to travel safely, preventive services and screenings recommended at different ages, where to go for reliable health information, and nutrition. The SA sessions did not include any PA topics. Additionally, it can be noted that the SA intervention included a 5 to 10 min instructor-led program of gentle upper extremity stretching or flexibility exercises.

### 2.3. Medication Use and Included Covariates

Medication use was assessed at baseline by visual inspection of all prescription and nonprescription medications taken in the previous two weeks as described previously [7,8]. A complete medication inventory was taken which included prescriptions, over-the-counter medications, vitamins, and supplements. Patients were grouped according to their summed anticholinergic drug burden at baseline defined using the Anticholinergic Cognitive Burden (ACB) scale [6,9]. The ACB scale measures probable anticholinergic activity on a scale from 0 to 3. A drug was given a score of 1 if it had possible anticholinergic effects based on lab tests but no evidence of clinically relevant cognitive effects, while a drug was given a score of 2 or 3 if it had established and clinically relevant anticholinergic effects. Medications not listed in the ACB scale were given a score of 0. ACB scores were derived for each participant by summing the score of each unique medication prescribed to generate an overall individual ACB score. Furthermore, anticholinergic burden was categorized into four groups: no burden (score = 0), possible burden (sum score = 1), low burden (sum score = 2), high burden (sum score = 3), very high burden (sum score = 4+). For example, a patient that was prescribed loperamide (score = 1) and amitriptyline (score = 3) would have a total summed score of 4 and furthermore be categorized into the very high burden grouping. 

Baseline data consisted of demographic information, medical history, medication inventory, body mass index, lower-extremity function measured via Short Physical Performance Battery (SPPB) [9,10]. Notably, factors that may be related to anticholinergic burden, such as cognitive assessment taken by the Modified Mini-Mental State Examination (3MSE), overall number of medications per participant, number of ACB medications per participant, feelings of dizziness in the previous six months, falls experienced in the previous year, and a fall requiring medical attention in the past year, were recorded. 

### 2.4. Follow-Up and Outcome Assessment 

Participants were assessed every six months at clinic visits. Home, telephone, and proxy assessments were attempted if participants could not come to the clinic. The assessment staff was blinded to the intervention and remained separate from the intervention team. Participants were asked not to disclose their assigned intervention arm or talk about their interventions during the assessment.

Details of MMD ascertainment were reported previously [11]. Briefly, participants were asked to walk 400 m at their usual pace, and MMD was defined as the inability to complete the walk within 15 min without sitting and without the help of another person or walker. When MMD could not be objectively measured because of the inability of the participant to come to the clinic and absence of a suitable walking course at the participant’s home, institution, or hospital, an alternative adjudication of the outcome was based on objective inability to walk 4 m in less than 10 s or self-, proxy-, or medical record-reported inability to walk across a room. If participants met these alternative criteria, they would not be able to complete the 400 m walk within 15 min. Two consecutive MMD assessments or MMD followed by death defined pMMD. Falls were assessed based on prior work [12], where the outcome of “injurious fall” was defined as a fall resulting in a fracture or injury requiring hospitalization. Outcomes were adjudicated by at least two reviewers using medical and hospital records.

### 2.5. Statistical Analysis

Participants were grouped to reflect sum ACB burden (e.g., no burden, possible burden, low burden, high burden, or very high burden) during the baseline assessment. Baseline characteristics were described and compared using ANOVA and chi-squared tests where appropriate for ACB burden categorization stratified by the intervention assignment and overall. MMD, pMMD, and injurious falls were assessed in proportional hazards regression models, and hazard ratios (HRs) and 95% confidence intervals (CIs) were reported. The proportional hazard assumption was confirmed using Schoenfeld residual plots. Several models were estimated either with the intervention and ACB burden grouping exposures as individual covariates or with an interaction effect between intervention and ACB burden grouping. Adjusted models included all measured baseline characteristics. All analyses were performed using SAS Enterprise Guide version 7.1 (SAS Institute, Cary, NC, USA).

## 3. Results

Of the 1635 participants in the LIFE study, 986 (60%) used at least one or more medication that was scored ≥1 by the ACB burden scale. A total of 463 (28.4%) participants had an ACB score equal to 1, 199 (12.2%) had an ACB summed score of 2, 156 (9.6%) an ACB summed score of 3, and 168 (10.3%) had an ACB summed score of 4+. Some of the most frequently taken ACB medications by participants included: atenolol, metoprolol, triamterene, furosimide, and warfarin (Table A1). Baseline characteristics of participants stratified by summed ACB score are in Table 1. Generally, those with higher ACB scores demonstrated more poor and fair self-rated health, less activity defined by minutes and steps active, and slower gait speed. Finally, the number of overall medications including those not considered to possess anticholinergic properties tended to increase with overall increasing ACB burden score. Notably the cognitive assessment (3MSE) demonstrated no significant differences between the groups. 

The number of crude events and follow-up time are reported by intervention arm (Table 2). Within the physical activity arm, those with an ACB score of 1 and those with an ACB score of 4+ demonstrated significantly more crude event rates for MMD, pMMD, and injurious falls than those with an ACB score of 0 (*p* < 0.05). Within the successful aging arm and MMD outcome, those with an ACB score of 1, 2, 3, and 4+ all had significantly more crude event rates than those with an ACB score of 0 (*p* < 0.05). For the successful aging arm and the pMMD outcome, those with an ACB score of 1, 2, and 3 all had significantly more crude event rates than those with an ACB score of 0 (*p* < 0.05). Finally, within the successful aging and injurious falls outcome, those with an ACB score of 4+ demonstrated significantly more crude event rates than those with an ACB score of 0 (*p* < 0.05). Crude event rates were only statistically significant between intervention arms within the MMD outcome at an ACB score of 3 (*p* < 0.05), and within the pMMD outcome at ACB scores of 2 and 3 (*p* < 0.05). 

**Figure 1:**
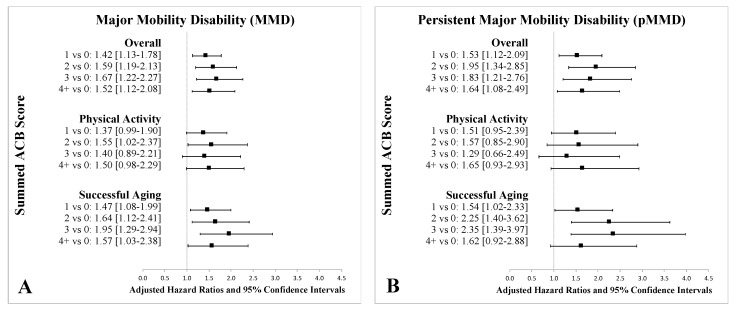

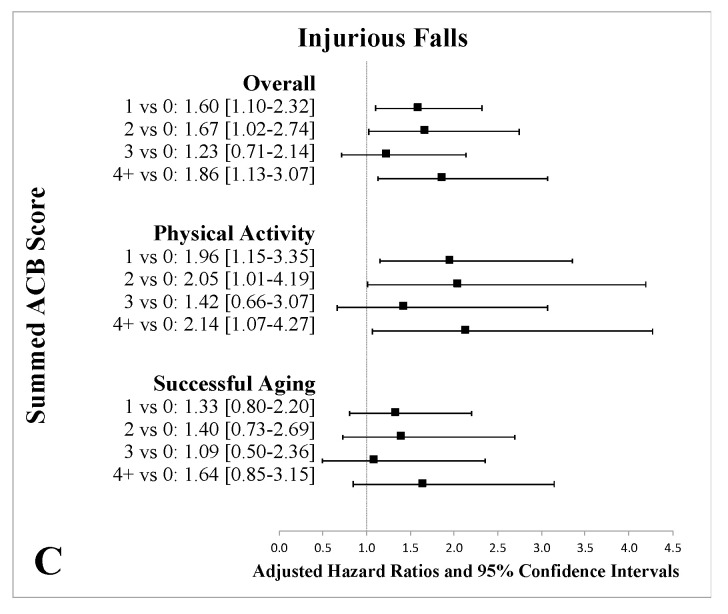
Adjusted Hazard Ratios and 95% Confidence Intervals for Major Mobility Disability (MMD) (**A**), Persistent Major Mobility Disability (pMMD) (**B**), and Injurious Falls (**C**) by Summed ACB Score and Intervention Arm. An odds ratio of >1 indicates higher risk for respective outcomes (MMD, pMMD, and injurious falls). Adjusted characteristics included: age, sex, race, education, systolic blood pressure, diastolic blood pressure, smoking status, body mass index, waist circumference, history of hypertension, history of stroke, history of diabetes, history of heart attack, history of heart failure, history of arthritis, history of chronic lung disease, history of cancer, short physical performance battery, self-rated overall health, activity levels, 400 m gait speed, cognitive assessment, overall number of medications, number of anticholinergic medications, patient experienced dizziness in past 6 months, patient experienced a fall in the past year, patient experienced a fall requiring medical attention in the past year.

Figure 1 shows the proportional hazard regression results. Compared to those with an ACB score of 0, individuals with an ACB score of 1 had a 42% increased rate of MMD (HR 1.42 [95% CI 1.13–1.78]), a 53% increased rate of pMMD (HR 1.53 [95% CI 1.12–2.09]), and a 60% increased rate of injurious falls (HR 1.60 [95% CI 1.10–2.32]). Results similar in magnitude were observed for all other ACB levels versus the no burden group (Figure 1). Those with an ACB score of 2 had a 59% increase of MMD (HR 1.59 [95% CI 1.19–2.13]), a 95% increase in pMMD (HR 1.95 [95% CI 1.34–2.85]), and a 67% increase in injurious falls (HR 1.67 [95% CI 1.02–2.74]) compared to those with an ACB score of 0. Those with an ACB score of 3 had a 67% increase of MMD (HR 1.67 [95% CI 1.22–2.27]), an 83% increase in pMMD (HR 1.83 [95% CI 1.21–2.76]), and no significant increase in injurious falls (HR 1.23 [95% CI 0.71–2.14]) compared to those with and ACB score of 0. Compared to those with an ACB score of 0, individuals with an ACB score of 4 had a 52% increase of MMD (HR 1.52 [95% CI 1.12–2.08]), a 64% increase in pMMD (HR 1.64 [95% CI 1.08–2.49]), and an 86% increase in injurious falls (HR 1.86 [95% CI 1.13–3.07]). Pairwise comparisons between groups with non-zero scores (i.e., 4+ vs. 3, 3 vs. 2, 2 vs. 1, etc.) did not demonstrate significant differences in any of the outcome measures.

Stratification of the proportional hazard regression results by PA or SA interventions also demonstrated few differences from the combined overall results (Figure 1). Results for the PA versus SA intervention were consistent with the prior findings of the LIFE study that demonstrated improved MMD and pMMD survival rates associated with the PA intervention.

## 4. Discussion

This analysis investigated the effect of participants taking medications with known anticholinergic properties on MMD, pMMD, and injurious falls outcomes in sedentary older adults participating in the LIFE study. All ACB levels (possible burden, low burden, high burden, very high burden) resulted in significant increases in MMD, pMMD, and injurious falls compared to those not taking these medications. The stratification of the intervention by ACB exposure demonstrated few differences from the combined overall results. Therefore, anticholinergic burden was not observed to potentially be associated with a negating effect of the LIFE Study intervention. 

There have been several published validated anticholinergic burden scales used to determine a patient’s overall anticholinergic burden including: Anticholinergic Risk Scale (ARS) [13], Anticholinergic Drug Scale (ADS) [14], and Drug Burden Index (DBI) [15], in addition to the Anticholinergic Cognitive Burden Scale (ACB) we utilized. There is poor agreement between the different anticholinergic scales in terms of measurement of anticholinergic activity as well as the medicines considered which makes comparisons between scales difficult [16]. The ARS was developed based upon an expert panel and categorical designation of cholinergic receptor dissociation levels on a three-point scale (none; 1, moderate; 2, strong; and 3, very strong) whereby the ARS score for a patient is the sum of points for his or her number of medications [13]. Similarly, the ADS was developed and validated using serum anticholinergic activity where drugs are ranked ordinally on a scale of 0 to 3 with zero signifying no known anticholinergic activity to 3 signifying marked anticholinergic activity [14]. Again, scores in this scale may be summed to determine a total score [14]. The DBI is somewhat different than the ARS and ADS in that it was created to measure total exposure to both anticholinergic and sedative medications and was developed using equations based upon recommended minimum daily doses approved by the US Food and Drug Administration [15]. Interestingly, with the development of the DBI, the exposure to medications with anticholinergic or sedating effects was associated with poorer performance on physical mobility and cognitive tasks in a cohort of older functioning adults [15]. DBI scoring is more complex than the ordinal numbering of ADS, ARS, and ACB scores in that all scores are on a logarithmic scale based upon daily dosing and then subsequently need to be summed to create an overall burden score. Furthermore, the DBI scale may not represent an easily employable method for identification of high anticholinergic burden in the community setting. 

We chose the ACB scale by Boustani et al. as this ordinal scale would represent a quick, clinically relevant way to quantify summed clinically relevant anticholinergic levels in a population of older adults. It should be noted that the ACB scale was developed by an expert panel whereby ACB drugs were ranked according to both in vitro anticholinergic activity levels as well as their established and clinically relevant cognitive effects. Additionally, previous, well-designed investigations have observed significant increases in cognitive decline and mortality in participants over two years using this scale [17]. Cumulatively, cognitive decline and mortality outcomes may be sensitive to overlap with our studied mobility outcomes MMD, pMMD, and injurious falls.

Stepwise comparisons (4 vs. 3, 3 vs. 2, 2 vs. 1) between different levels of ACB did not demonstrate significant differences for MMD, pMMD, or injurious falls. This indicates that the LIFE participants did not demonstrate a dose–response relationship in the level of anticholinergic burden on our tested outcomes. Although we do not observe a dose response with higher ACB scores, the effect is consistent in that ACB medications confer a higher risk of MMD, pMMD, and injurious falls. It is important to note that although the ACB scale indicates a delineation of scores between ACB medications, the scale uses a point system based upon level of evidence and not necessarily level of effect. These results suggest that the ACB scale, and potentially other existing anticholinergic burden tools, lack sensitivity to distinguish changes in these physical functioning outcomes with increases in anticholinergic burden. Future studies should aim to establish the validity of various drug burden scales for this purpose. 

The direct effects of ACB medications on our studied outcomes could potentially be mediated through both physical and cognitive pathways. Although baseline cognitive function measured by 3MSE was not different between ACB groupings, previous studies have found that anticholinergic medications were associated with worse balance and more falls [18]. This has been speculated to occur due to a reduction in processing speed and promotion of cerebral vasculopathy and consequent white matter changes [19]. In line with our results, other studies have found that anticholinergic burden is associated with greater decline in objectively measured physical activity over time in older adults [19]. Additionally, ACB has been associated with a higher incidence of cardiovascular events, which may also demonstrate declines in mobility [20].

A recent prospective cohort study in Canada evaluated baseline use of anticholinergic or sedative medications as measured by the drug burden index and found that there was a significant association between baseline burden of exposure and functional status decline at six months as measured by the Older Americans Resources and Services score (OARS) [21]. In line with this cohort study, the LIFE trial demonstrated significant increases in MMD, pMMD, and injurious falls in those carrying baseline anticholinergic burdens. Although the LIFE trial only measured outcomes of mobility and not a broad functional status, this study adds to the evolving evidence of the broad deleterious effects of anticholinergic medications on both physical function and holistic functional status of older adults. 

There were limitations to this study including the fact this was a post hoc subgroup analysis of the LIFE study, which was not specifically powered to detect the influence of medications on outcomes. Additionally, this study ascertained medication exposure information only at baseline and this initial classification may not represent consistent anticholinergic exposure throughout the entire follow-up period as the medication use process is generally dynamic over time. Moreover, participants were not randomized to anticholinergic burden groups although many baseline measures were similar between these medication users. Adjustment techniques were used on measured baseline covariates, however, the potential for residual confounding cannot be ruled out. Stepwise comparisons between different levels of ACB did not demonstrate significant differences in survival in MMD, pMMD, or injurious falls. This may be due to insufficient power considering the smaller number of individuals reporting higher ACBs. 

## 5. Conclusions

This analysis raises questions about the effect of medications with anticholinergic burden on mobility as well as the ability to preserve mobility through the implementation of regular physical activity and lifestyle interventions. We showed a strong association between those using medications with anticholinergic properties and mobility disability outcomes and injurious falls when compared to those not using these medications. Dose response or stepwise comparisons between ACB groupings did not demonstrate significant differences in survival in MMD, pMMD, or injurious falls. Clinicians should consider the necessity or deprescribing of medications with known anticholinergic properties because they may have unintended, detrimental consequences on mobility. Further investigations are required to establish a scale that can capture increasing anticholinergic burden and effects on mobility and physical independence. 

## Figures and Tables

**Table 1:** Baseline Characteristics of Lifestyle Intervention for Elders (LIFE) Participants by Total Anticholinergic Burden Score.

Characteristics	Summed Total Anticholinergic Burden Score					*p*-Value
	ACB Score = 0 *n* = 644 (39.5%)	ACB Score = 1 *n* = 463 (28.4%)	ACB Score = 2 *n* = 199 (12.2%)	ACB Score = 3 *n* = 156 (9.6%)	ACB Score = 4+ *n* = 168 (10.3%)	
Age, Mean (SD)	78.7 (5.2)	79.2 (5.0)	78.9 (5.5)	78.8 (5.2)	78.9 (5.4)	0.6440
Female	446 (69.3%)	298 (64.4%)	123 (61.8%)	100 (64.1%)	127 (75.6%)	0.0208
Race						
White	499 (77.5%)	335 (72.4%)	148 (74.4%)	123 (78.9%)	137 (81.6%)	0.1831
Black	105 (16.3%)	98 (21.2%)	40 (20.1%)	21 (13.5%)	21 (12.5%)	
Other	40 (6.2%)	30 (6.5%)	11 (5.3%)	12 (7.7%)	10 (6.0%)	
Education, ≥High School	434 (67.4%)	289 (62.4%)	125 (62.8%)	99 (63.5%)	100 (59.5%)	0.2583
Systolic Blood Pressure, Mean (SD)	127.2 (17.5)	127.7 (19.0)	129.0 (18.1)	125.6 (18.0)	127.7 (16.4)	0.4832
Diastolic Blood Pressure, Mean (SD)	68.6 (10.0)	67.5 (10.6)	68.6 (9.8)	68.1 (10.2)	68.2 (10.2)	0.4007
Smoking Status						
Never	343 (53.3%)	242 (52.3%)	109 (54.8%)	78 (50.0%)	88 (52.4%)	0.9125
Former	279 (43.3%)	205 (44.3%)	86 (43.2%)	74 (47.4%)	77 (45.8%)	
Current	22 (3.4%)	16 (3.5%)	4 (2.0%)	4 (2.6%)	3 (1.8%)	
Body Mass Index (SD)	29.7 (5.7)	30.2 (5.9)	30.9 (6.3)	31.0 (6.4)	30.5 (6.4)	0.0517
Average Waist Circumference in cm (SD)	100.8 (15.6)	101.9 (15.2)	104.0 (15.2)	103.6 (15.0)	101.0 (16.0)	0.0624
Hypertension	378 (58.7%)	358 (77.3%)	165 (82.9%)	111 (71.2%)	139 (82.7%)	<0.0001
Stroke	41 (6.4%)	33 (7.1%)	19 (9.6%)	6 (3.9%)	10 (6.0%)	0.2850
Diabetes	138 (21.4%)	117 (25.3%)	61 (30.7%)	43 (27.6%)	56 (33.3%)	0.0067
Heart Attack	25 (3.9%)	44 (9.5%)	30 (15.1%)	8 (5.1%)	22 (13.1%)	<0.0001
Heart Failure	9 (1.4%)	19 (4.1%)	12 (6.0%)	11 (7.1%)	20 (11.9%)	<0.0001
Arthritis	114 (17.7%)	89 (19.2%)	45 (22.6%)	31 (19.9%)	39 (23.2%)	0.3971
Chronic Lung Disease	88 (13.7%)	63 (13.6%)	37 (18.6%)	32 (20.5%)	33 (19.6%)	0.0573
Cancer	125 (19.4%)	113 (24.4%)	42 (21.1%)	42 (11.4%)	48 (13.0%)	0.0419
SPPB ≤ 7	379 (58.9%)	255 (55.1%)	110 (55.3%)	78 (50.0%)	79 (47.0%)	0.0454
Self-Rated Overall Health						
Poor	7 (1.1%)	7 (1.5%)	2 (1.0%)	0 (0.0%)	4 (2.4%)	<0.0001
Fair	79 (12.3%)	71 (15.3%)	36 (18.1%)	31 (19.9%)	34 (20.2%)	
Good	276 (42.9%)	240 (51.8%)	114 (57.3%)	78 (50.0%)	87 (51.8%)	
Very Good	228 (35.4%)	119 (25.7%)	38 (19.1%)	41 (9.0%)	36 (21.4%)	
Excellent	54 (8.4%)	26 (5.6%)	9 (4.5%)	6 (3.9%)	7 (4.2%)	
Moderate Activity (Daily Average), Mean (SD)						
Minutes	32 (28)	24 (21)	26 (22)	25 (20)	21 (15)	<0.0001
Steps	1162 (1245)	869 (920)	975 (1116)	797 (833)	720 (752)	<0.0001
400 Meter Gait Speed (meters/second), Mean (SD)	0.77 (0.16)	0.76 (0.16)	0.78 (0.17)	0.74 (0.14)	0.74 (0.16)	0.0230
Cognitive Assessment (3MSE), Mean (SD)	92.0 (5.3)	91.4 (5.4)	91.1 (5.7)	91.5 (5.8)	91.5 (5.3)	0.1872
Overall Number of Medications, Mean (SD)	7.6 (3.9)	9.1 (3.8)	10.9 (3.9)	10.5 (4.3)	13.3 (4.8)	<0.0001
Number of ACB Medications, Mean (SD)	0 (0)	1 (0)	1.97 (0.2)	1.8 (1.0)	3.0 (1.0)	<0.0001
Experienced Dizziness in Past 6 Months	137 (21.3%)	76 (16.4%)	48 (24.1%)	32 (20.5%)	50 (29.8%)	0.0051
Experienced Fall in Past Year	317 (49.2%)	213 (46.0%)	95 (47.7%)	93 (59.6%)	98 (58.3%)	0.0074
Experienced Fall Requiring Medical Attention (Past Year)	74 (11.5%)	53 (11.5%)	25 (12.6%)	16 (10.3%)	21 (12.5%)	0.9625

Abbreviations: ACB (Anticholinergic Cognitive Burden); 3MSE (Modified Mini-Mental State Examination); SD (Standard Deviation); SPPB (Short Physical Performance Battery); HRQL (Health-Related Quality of Life).

**Table 2:** Unadjusted Number of Events, Person-Years, and Event Rates for Major Mobility Disability, Persistent Major Mobility Disability, and Injurious Falls by Anticholinergic Cognitive Burden Summed Score and Intervention Arm.

	Physical Activity			Successful Aging		
Outcome	Events, *n*	Person-Years (PY)	Events/100 PY	Events, *n*	Person-Years (PY)	Events/100 PY
**MMD**						
ACB score = 0	78	759.80	10.26	87	724.66	12.01
ACB score = 1	73	494.3	14.77 *	88	519.2	16.95 *
ACB score = 2	33	216.99	15.21	43	208.39	20.63 *
ACB score = 3	25	174.7	14.31	37	138.79	26.66 *^,†^
ACB score= 4+	36	176.4	20.41 *	35	178.84	19.57 *
**pMMD**						
ACB score = 0	36	782.03	4.60	46	754.38	6.10
ACB score = 1	39	510.83	7.63 *	49	534.9	9.16 *
ACB score = 2	16	212.26	7.54	32	216.03	14.81 *^,†^
ACB score = 3	12	183.62	6.54	24	144.37	16.62 *^,†^
ACB score = 4+	21	186.99	11.23 *	18	180.89	9.95
**Injurious Falls**						
ACB score = 0	27	831.84	3.25	33	801.41	4.12
ACB score = 1	31	555.16	5.58 *	31	601.38	5.15
ACB score = 2	12	237.07	5.06	14	260.16	5.38
ACB score = 3	9	200.22	4.50	9	181.93	4.95
ACB score = 4+	16	209.72	7.63 *	15	206.53	7.26 *

Abbreviations: ACB (Anticholinergic Cognitive Burden); MMD (Major Mobility Disability); pMMD (Persistent Major Mobility Disability). * Chi-square comparison to ACB score = 0 within each intervention arm (indicative of *p* < 0.05). ^†^ Chi-square comparison between intervention within each ACB score (indicative of *p* < 0.05).

## Appendix A

**Table A1:** Baseline Anticholinergic Medication Prevalence by Anticholinergic Cognitive Burden (ACB) Score of the Lifestyle Interventions for Elders (LIFE) Participants.

Score 1	Score 2	Score 3
Alimemazine; *n* = 0	Amantadine; *n* = 0	Amitriptyline; *n* = 27
Alverine; *n* = 0	Belladone alkaloids; *n* = 1	Amoxapine; *n* = 0
Alprazolam; *n* = 39	Carbamazepine; *n* = 4	Atropine; *n* = 7
Atenolol; *n* = 201	Cyclobenzaprine; *n* = 14	Benztropine; *n* = 0
Bupropion; *n* = 24	Cyproheptadine; *n* = 0	Brompheniramine; *n* = 0
Captopril; *n* = 3	Empracet; *n* = 0	Carbinoxamine; *n* = 0
Chlorthalidone; *n* = 20	Loxapine; *n* = 0	Chlorpheniramine; *n* = 14
Cimetidine; *n* = 3	Meperidine; *n* = 0	Chlorpromazine; *n* = 0
Ranitidine; *n* = 57	Methotrimeprazine; *n* = 0	Clemastine; *n* = 0
Clorazepate; *n* = 1	Molindone; *n* = 0	Clomipramine; *n* = 0
Codeine; *n* = 11	Oxcarbazepine; *n* = 4	Clozapine; *n* = 0
Colchicine; *n* = 17	Pethidine hydrochloride; *n* = 0	Darifenacin; *n* = 10
Coumadin; *n* = 163	Pimozide; *n* = 0	Desipramine; *n* = 2
Diazepam; *n* = 8		Dicyclomine; *n* = 3
Digoxin; *n* = 57		Dimenhydrinate; *n* = 0
Dipyridamole; *n* = 4		Diphenhydramine; *n* = 26
Disopyramide; *n* = 1		Doxepin; *n* = 1
Fentanyl; *n* = 2		Flavoxate; *n* = 0
Furosemide; *n* = 173		Hydroxyzine; *n* = 9
Fluvoxamine; *n* = 0		Hyoscyamine; *n* = 3
Haloperidol; *n* = 1		Imipramine; *n* = 6
Hydralazine; *n* = 22		Meclizine; *n* = 19
Hydrocortisone; *n* = 13		Nortriptyline; *n* = 4
Isosorbide; *n* = 42		Olanzapine; *n* = 1
Loperamide; *n* = 28		Orphenadrine; *n* = 0
Metoprolol; *n* = 301		Oxybutynin; *n* = 45
Morphine; *n* = 3		Paroxetine; *n* = 26
Nifedipine; *n* = 40		Perphenazine; *n* = 1
Prednisone; *n* = 37		Procyclidine; *n* = 0
Quinidine; *n* = 2		Promazine; *n* = 0
Risperidone; *n* = 2		Promethazine; *n* = 1
Theophylline; *n* = 4		Propantheline; *n* = 0
Trazodone; *n* = 26		Pyrilamine; *n* = 0
Triamterene; *n* = 72		Tolterodine; *n* = 28
		Quetiapine; *n* = 2
